# Inflammatory Burden Shapes Antioxidant Adaptation and Immunometabolic Remodeling in LPS-Activated BV-2 Microglial Cells

**DOI:** 10.3390/ijms27208981

**Published:** 2026-10-10

**Authors:** Maria Cristina Barbalace, Irene Rinaldi, Martina Mantini, Lorenzo Zallocco, Maurizio Ronci, Federica Di Cintio, Alessandro Attanzio, Cecilia Prata, Marco Malaguti, Silvana Hrelia, Laura Giusti, Cristina Angeloni

**Affiliations:** 1 Department for Life Quality Studies, Alma Mater Studiorum, University of Bologna, Corso d’Augusto 237, 47921 Rimini, Italy; irene.rinaldi5@unibo.it (I.R.); martina.mantini2@unibo.it (M.M.); marco.malaguti@unibo.it (M.M.); silvana.hrelia@unibo.it (S.H.); cristina.angeloni@unibo.it (C.A.); 2 Department of Translational Research and New Technologies in Medicine and Surgery, University of Pisa, 56126 Pisa, Italy; l.zallocco@gmail.com; 3 IRCCS Stella Maris Foundation, Viale del Tirreno 331, Calambrone, 56128 Pisa, Italy; 4 Department of Medical, Oral and Biotechnological Sciences, University G. D’Annunzio of Chieti-Pescara, 66100 Chieti, Italy; maurizio.ronci@unich.it (M.R.); federica.dicintio@unich.it (F.D.C.); 5 Department of Biological, Chemical and Pharmaceutical Sciences and Technologies, University of Palermo, Via Archirafi 28, 90123 Palermo, Italy; alessandro.attanzio@unipa.it; 6 Department of Pharmacy and Biotechnology, Alma Mater Studiorum, University of Bologna, Via Irnerio, 42, 40126 Bologna, Italy; cecilia.prata@unibo.it

**Keywords:** microglia, BV-2, neuroinflammation, oxidative stress, antioxidant enzymes, proteomics, lipopolysaccharide, clathrin-mediated endocytosis, lysosomal activity

## Abstract

Neuroinflammation is a hallmark of numerous neurodegenerative diseases and is characterized by profound inflammatory, metabolic, and redox alterations in activated microglia. Although lipopolysaccharide (LPS)-stimulated BV-2 cells represent one of the most widely used in vitro models of neuroinflammation, a comprehensive understanding of the coordinated molecular adaptations underlying this model remains incomplete. The aim of this study was to characterize the proteomic remodeling of BV-2 cells exposed to LPS 100 ng/mL for 24 h and investigate the relationship between antioxidant and inflammatory responses across increasing levels of LPS exposure. Proteomic analysis identified 89 differentially expressed proteins involved in cytoskeletal organization, clathrin-mediated endocytosis, lysosomal function, immunometabolism, and redox regulation. Validation experiments confirmed changes in cytoskeletal and endocytic proteins, including Vimentin, Gelsolin, Disabled homolog-2, and clathrin light chain A, together with modulation of proteins involved in the lysosomal pathways and marked induction of the immunometabolic enzyme aconitate decarboxylase 1. Functional enrichment analysis identified Nuclear factor erythroid 2-related factor 2 (NRF2) as the most significantly activated upstream regulator, a prediction consistent with engagement of an adaptive antioxidant program. Interestingly, analysis of increasing LPS concentrations revealed that antioxidant responses were not proportional to the inflammatory stimulus. Expression of NRF2-controlled antioxidant enzymes progressively increased for LPS concentrations up to 500 ng/mL, but declined at 1000 ng/mL LPS, whereas pro-inflammatory mediators remained continuously upregulated. This pattern is consistent with the attenuation of antioxidant adaptation under the highest inflammatory burden tested. Together, these findings demonstrate that the BV-2/LPS model is characterized by a dynamic interplay between inflammatory, redox, metabolic, lysosomal, endocytic, and cytoskeletal pathways, providing a more comprehensive characterization to support the interpretation and experimental use of this neuroinflammatory model.

## 1. Introduction

Neuroinflammation, the inflammatory response within the central nervous system (CNS), is a complex and dynamic process that exerts both beneficial and harmful effects, depending on the nature, duration, and context of the stimulus. While acute and tightly regulated neuroinflammation supports CNS homeostasis and tissue repair, chronic or dysregulated neuroinflammatory responses are now recognized as pivotal contributors to the progression of neurodegeneration [1,2].

Microglia, the resident immune cells of the CNS, are the principal mediators of the innate immune response in the brain. Under physiological conditions, microglia adopt a surveillant phenotype, constantly monitoring the brain microenvironment [3]. Upon stimulation by pathogens, injury, or endogenous danger signals, microglia rapidly shift toward an activated phenotype, characterized by morphological changes and the secretion of inflammatory mediators such as tumor necrosis factor-alpha (TNF-α), interleukin-1 beta (IL-1β), inducible nitric oxide synthase (iNOS), and reactive oxygen species (ROS) [4]. While this response is crucial for limiting damage and initiating repair, persistent microglial activation has been linked to synaptic dysfunction, neuronal loss, and the chronic progression of neurodegenerative processes [5].

Lipopolysaccharide (LPS) remains the most widely used experimental stimulus for investigating microglial inflammatory activation in vitro. Through binding to the CD14/MD2/TLR4 receptor complex, LPS activates multiple intracellular signaling pathways, including NF-κB, ultimately promoting the expression of pro-inflammatory mediators such as TNF-α, IL-1β, iNOS, and cyclooxygenase-2 (COX-2) [6,7]. Among the downstream consequences of LPS stimulation, oxidative stress is a major determinant of microglial function. Increased production of ROS and reactive nitrogen species (RNS), resulting from activation of NADPH oxidase, iNOS, and mitochondrial dysfunction, amplifies inflammatory signaling and contributes to neuronal injury [8,9,10,11,12]. To counteract oxidative damage, cells activate the transcription factor nuclear factor erythroid 2-related factor 2 (NRF2), which regulates numerous antioxidant and cytoprotective genes, including heme oxygenase-1 (HO-1) and NAD(P)H: quinone oxidoreductase-1 (NQO1) [13]. However, despite the recognized interplay between inflammation and oxidative stress, it remains unclear how antioxidant adaptation evolves as inflammatory activation becomes progressively more severe.

The murine BV-2 cell line, originally developed by Blasi et al. [14], is widely employed as a reproducible experimental model for mechanistic studies of microglial responses to inflammatory stimuli due to its ease of culture and phenotypic similarities to native microglia, although they do not fully recapitulate the phenotype and functional complexity of primary microglia [15,16].

Recent applications of this model include the use of BV-2 cells exposed to LPS to monitor activation-associated cellular changes through live-cell imaging [17]. Moreover, we have extensively employed LPS-stimulated BV-2 cells to evaluate the anti-neuroinflammatory activity of nutraceutical compounds [18,19,20,21,22]. Previous proteomic studies have substantially contributed to the characterization of LPS-induced microglial activation by identifying proteins and pathways associated with inflammatory stimulation [16,21,23,24,25]. Although these studies have demonstrated that LPS profoundly alters the microglial proteome, less attention has been devoted to linking these proteomic alterations to the functional remodeling of the activated phenotype.

In the present study, we first combined proteomic profiling with targeted molecular analyses to characterize the remodeling of BV-2 cells exposed to a predefined reference condition of 100 ng/mL LPS for 24 h. This condition was selected for the proteomic analysis because it is widely used to induce a robust and reproducible inflammatory phenotype in BV-2 microglial cells, thereby facilitating comparison with previous studies employing this experimental model [17,21,26]. In separate concentration-response experiments, we then examined cellular metabolic activity, oxidative status, antioxidant adaptation, and inflammation. This two-stage design was intended to characterize the reference BV-2/LPS model and to determine whether increasing LPS exposure was associated with different antioxidant and inflammatory response patterns.

## 2. Results

### 2.1. Differential Protein Espression Analysis

**Figure 1:**
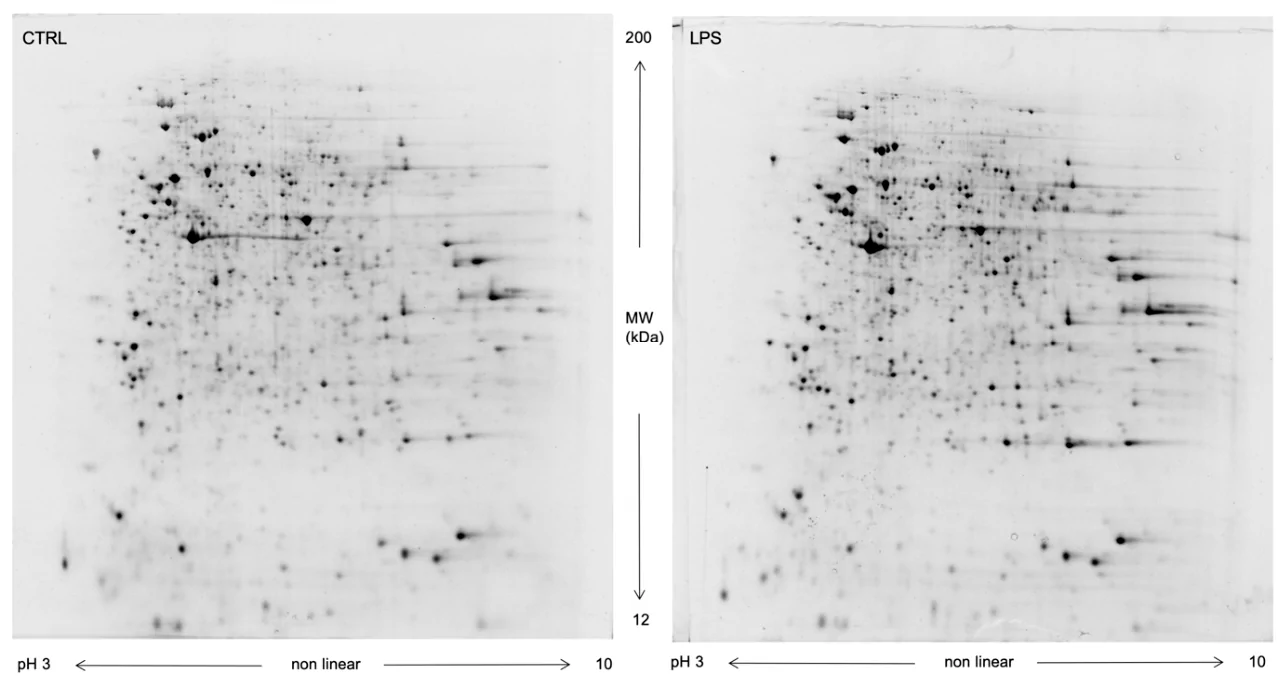
Representative 2D gel maps of protein profiles of BV-2 cells before (CTRL) and after LPS treatment (LPS). A total of 200 µg of proteins was separated using a linear pH gradient ranging from 3 to 10. The second dimension was performed by 12% sodium dodecyl sulfate–polyacrylamide gel electrophoresis (SDS–PAGE). Gels were stained with a fluorescent dye.

**Figure 2:**
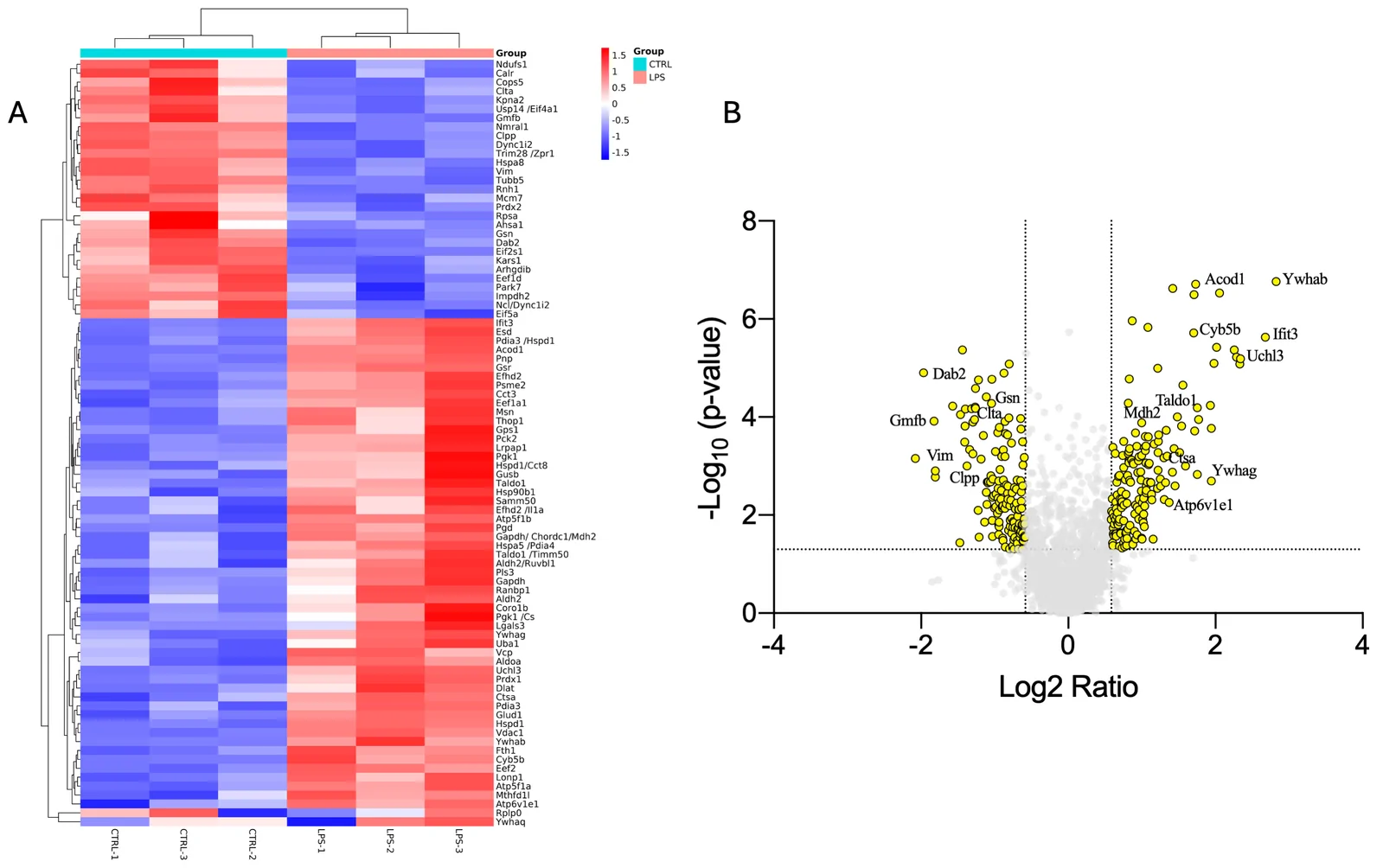
Hierarchically clustered heat map (**A**) and Volcano plot (**B**) obtained by comparative analysis between LPS and CTRL protein profiles. (**A**) The heat map was generated using SRplot software. Red and blue indicate increased and decreased protein expression, respectively, with color intensity reflecting the magnitude of the change. (**B**) Scatter plot of log2 fold change (LPS/CTRL) on the x-axis versus −log10 *p*-value on the y-axis for quantified proteins obtained from the comparison between LPS and CTRL protein profiles. Upregulated and downregulated proteins are shown on the right (Log2ratio ≥ +0.584) and on the left (Log2 ratio ≤ −0.584), respectively, while non-significant proteins are shown in grey. Dotted lines indicate the significance threshold (*p* < 0.05) and the fold change cut-off (fold change ≥ 1.5). Protein gene names correspond to the identifications reported in the Supplementary Table S1. Gene name of proteins is shown.

Representative two-dimensional protein profiles of BV-2 cells before and after LPS treatment are shown in Figure 1. One hundred and one protein spots were found to be differentially expressed after LPS treatment. Among these, 66 were increased, and 35 were decreased with *p*-value < 0.05 and fold change ≥ 1.5. These 101 differentially expressed spots were identified by LC-MS/MS and collapsed into 89 proteins because some proteins were identified in multiple spots. Spot-level intensities were not summed or averaged, as different spots containing the same protein may represent distinct proteoforms or post-translationally modified species. The names of identified proteins, the molecular weight (MW), isoelectric point (pI), score, coverage values of MS/MS, ratio, *p*-values, and *q*-value are listed in Supplementary Table S1. In Figure 2A, a heatmap depicts the differentially expressed proteins, whereas the Volcano plot (Figure 2B) graphically represents fold changes in quantified proteins obtained for the LPS vs. CTRL comparison.

Analysis of protein–protein interactions was conducted using Ingenuity Pathway Analysis. Upstream regulators with significant z-scores are listed in Table 1. Among these, the NRF2 transcription regulator, encoded by the *Nfe2l2* gene, was the most significantly activated factor, with a z-score of 2.917 and a *p*-value of 2.74 × 10^−17^, and 22 proteins correlated with it. Activation of this regulator is consistent with a compensatory response aimed at limiting LPS-induced damage. The analysis also predicted the activation of interleukin-15 (IL-15), encoded by the *Il15* gene, with an activation z-score of 2.02. Unlike the cytoprotective role attributed to NRF2, predicted IL-15 activation reinforces pro-inflammatory signals in the context of LPS-induced neuroinflammation of microglial cells. Proteins identified in multiple spots, including Peroxiredoxin-1 (PRDX1), 60S acidic ribosomal protein P0 (RPLP0), Tubulin beta-5 chain (TUBB5), Protein disulfide-isomerase A3 (PDIA3), Phospho-glycerate kinase 1 (PGK1), and 14-3-3 protein theta (YWHAQ), may represent distinct proteoforms or post-translationally modified species. Therefore, their spot-level fold changes were not interpreted as changes in total protein abundance, and their contribution to the predicted upstream-regulator was considered exploratory.

### 2.2. Validation of Proteomic Results

A subset of differentially expressed proteins was selected for targeted follow-up according to biological priority. Corresponding transcripts were measured by real-time PCR, and selected proteins were independently assessed by immunoblotting according to assay feasibility. BV-2 cells were treated with 100 ng/mL LPS for 3 h and 24 h for gene expression analysis or 24 h for protein level determination.

Among the differentially expressed proteins, two regulators of cytoskeleton dynamics, namely Vimentin (VIM) and Gelsolin (GSN), were identified as down-regulated after 24 h of LPS exposure.

**Figure 3:**
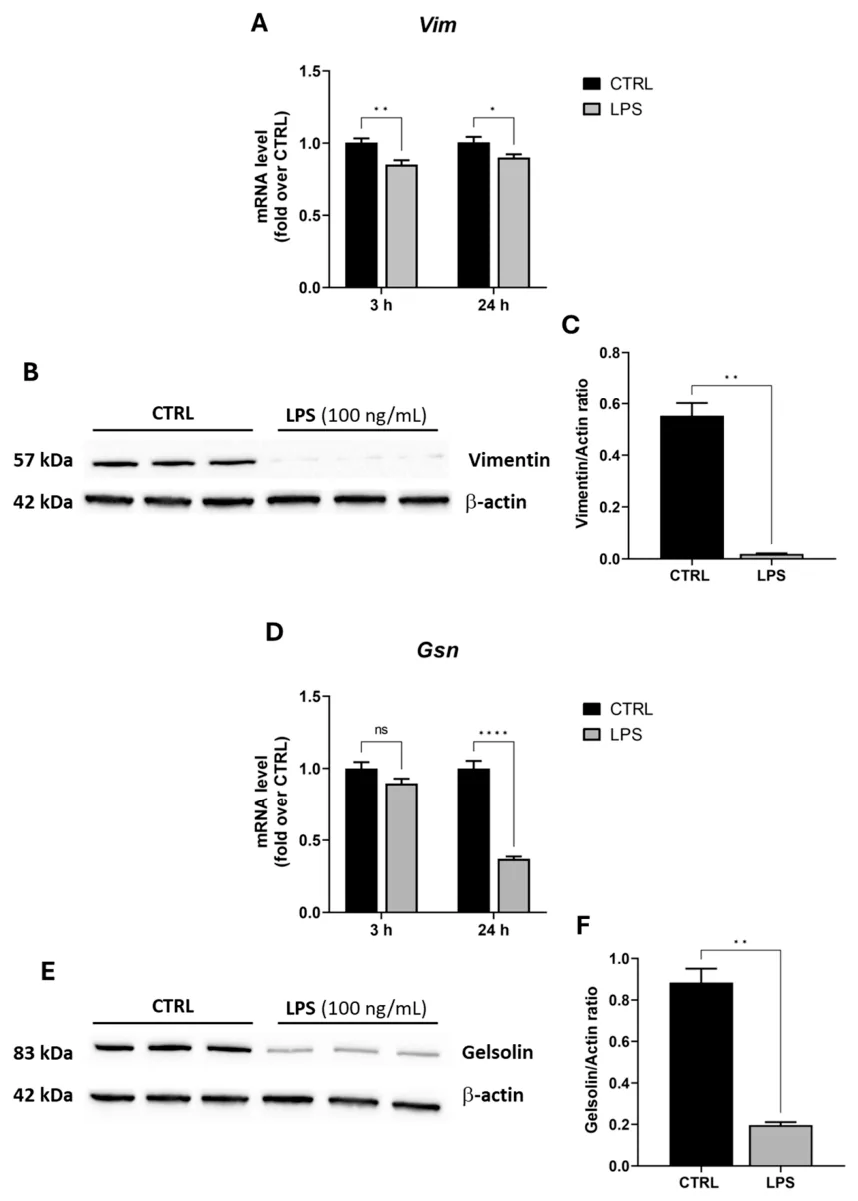
*Vim* and *Gsn* gene expression and VIM and GSN protein abundance. BV-2 cells were treated with 100 ng/mL LPS for 3 h (**A**,**D**) and/or 24 h (**A**,**B**,**D**,**E**). mRNA and protein levels were measured by real-time PCR and immunoblotting, respectively. Data are presented as mean ± SEM of at least three independent biological experiments performed on separate days (Table S2). Statistical analysis was performed using a two-way ANOVA, followed by Sidak’s post hoc test (**A**–**D**), and an unpaired *t*-test with Welch’s correction (**C**–**F**). * *p* < 0.05; ** *p* < 0.01; **** *p* < 0.0001. ns not significant.

As shown in Figure 3A, exposure to LPS resulted in a significant reduction in *Vim* mRNA levels at both 3 h and 24 h compared with control cells. Immunoblotting confirmed a marked reduction in VIM protein abundance after 24 h (Figure 3B,C), consistent with the direction of the proteomic change and *Vim* mRNA levels.

At the same time, *Gsn* mRNA and GSN protein levels were significantly reduced compared to control cells at 24 h (Figure 3D–F). Notably, LPS did not significantly alter *Gsn* mRNA levels after 3 h of incubation, indicating that this effect occurs at a later stage of microglial activation.

Proteomic analysis also identified the downregulation of disabled homolog 2 (DAB2) and clathrin light chain A (CLTA), two proteins associated with clathrin-mediated endocytosis (CME) and involved in receptor internalization and clathrin-coated vesicle formation, respectively.

**Figure 4:**
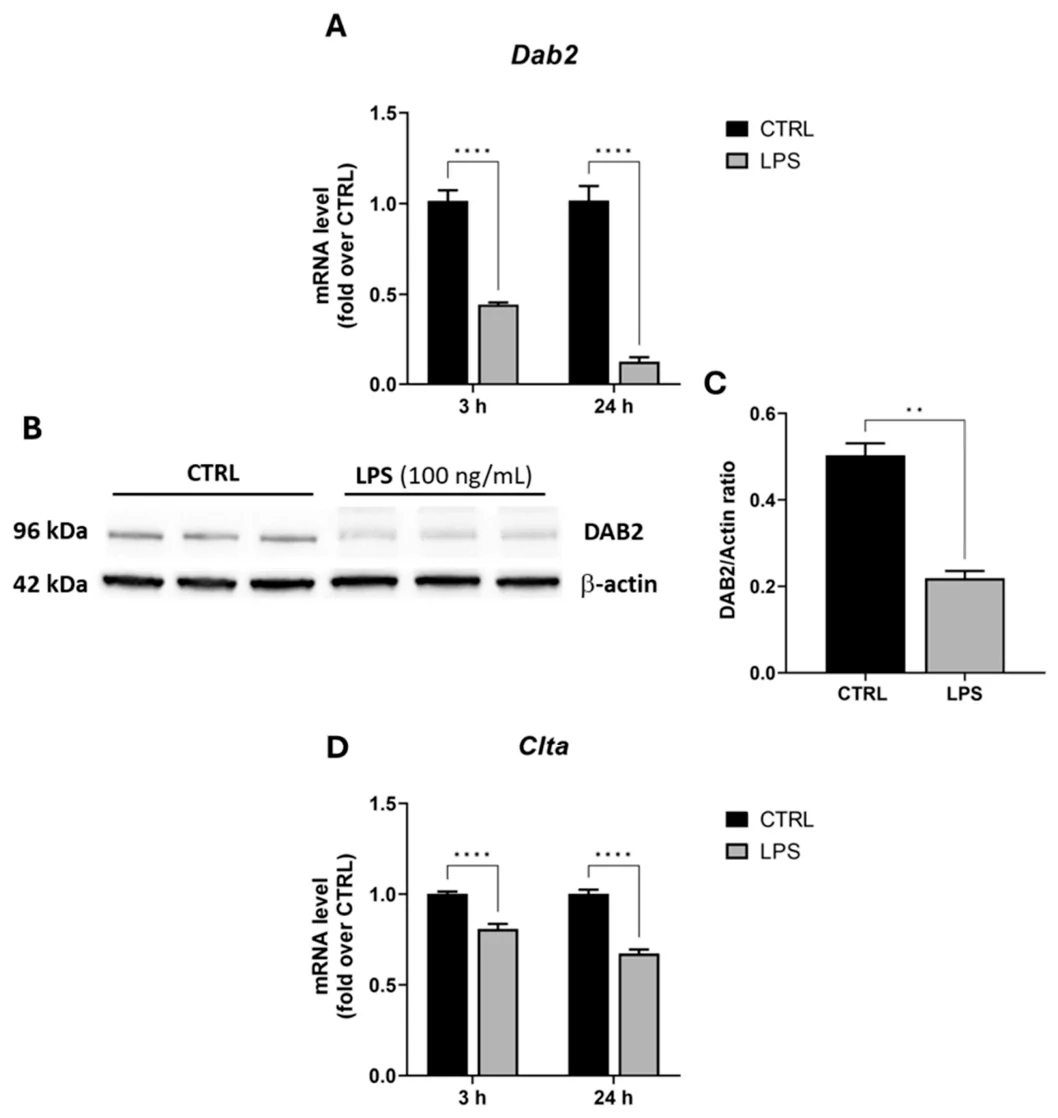
*Dab2* and *Clta* gene expression and DAB2 protein abundance. BV-2 cells were treated for 3 h (**A**,**D**) and/or 24 h (**A**,**B**,**D**) with 100 ng/mL LPS. mRNA and protein levels were measured by real-time PCR and immunoblotting, respectively. Data are presented as mean ± SEM of at least three independent biological experiments performed on separate days (Table S2). Statistical analysis was performed using a two-way ANOVA, followed by Sidak’s post hoc test (**A**–**D**), and an unpaired *t*-test with Welch’s correction (**C**). ** *p* < 0.01; **** *p* < 0.0001.

*Dab2* mRNA levels were significantly reduced after both 3 h and 24 h of LPS exposure, and DAB2 protein abundance was reduced after 24 h (Figure 4A–C). *Clta* mRNA was also significantly reduced at 3 h and 24 h (Figure 4D), consistent with the proteomic change observed at 24 h.

**Figure 5:**
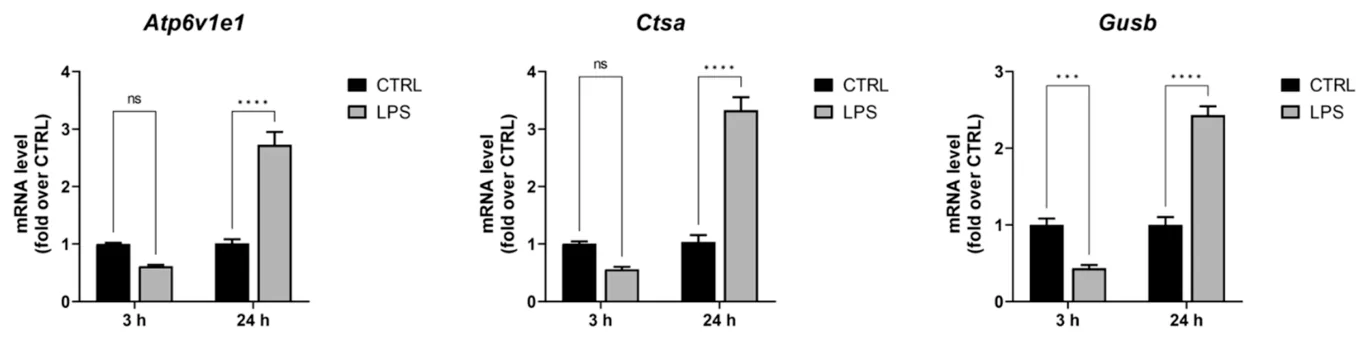
Expression of *Atp6v1e1*, *Ctsa*, and *Gusb* in BV-2 cells. BV-2 cells were treated with 100 ng/mL LPS for 3 h and 24 h. mRNA levels were measured by real-time PCR as reported in Materials and Methods. Data are presented as mean ± SEM of at least three independent biological experiments performed on separate days (Table S2). Statistical analysis was performed using a two-way ANOVA, followed by Sidak’s post hoc test. *** *p* < 0.001; **** *p* < 0.0001. ns not significant.

Moreover, the proteomic dataset showed increased expression of the lysosome-associated proteins ATPase H+ transporting V1 subunit E1 (ATP6V1E1), cathepsin A (CTSA), and beta-glucuronidase (GUSB) after 24 h of LPS exposure. Their corresponding transcripts (*Atp6v1e1*, *Ctsa*, and *Gusb*) were markedly up-regulated at 24 h. At 3 h, *Atp6v1e1* and *Ctsa* mRNA levels did not significantly differ from control, whereas *Gusb* expression was reduced (Figure 5).

**Figure 6:**
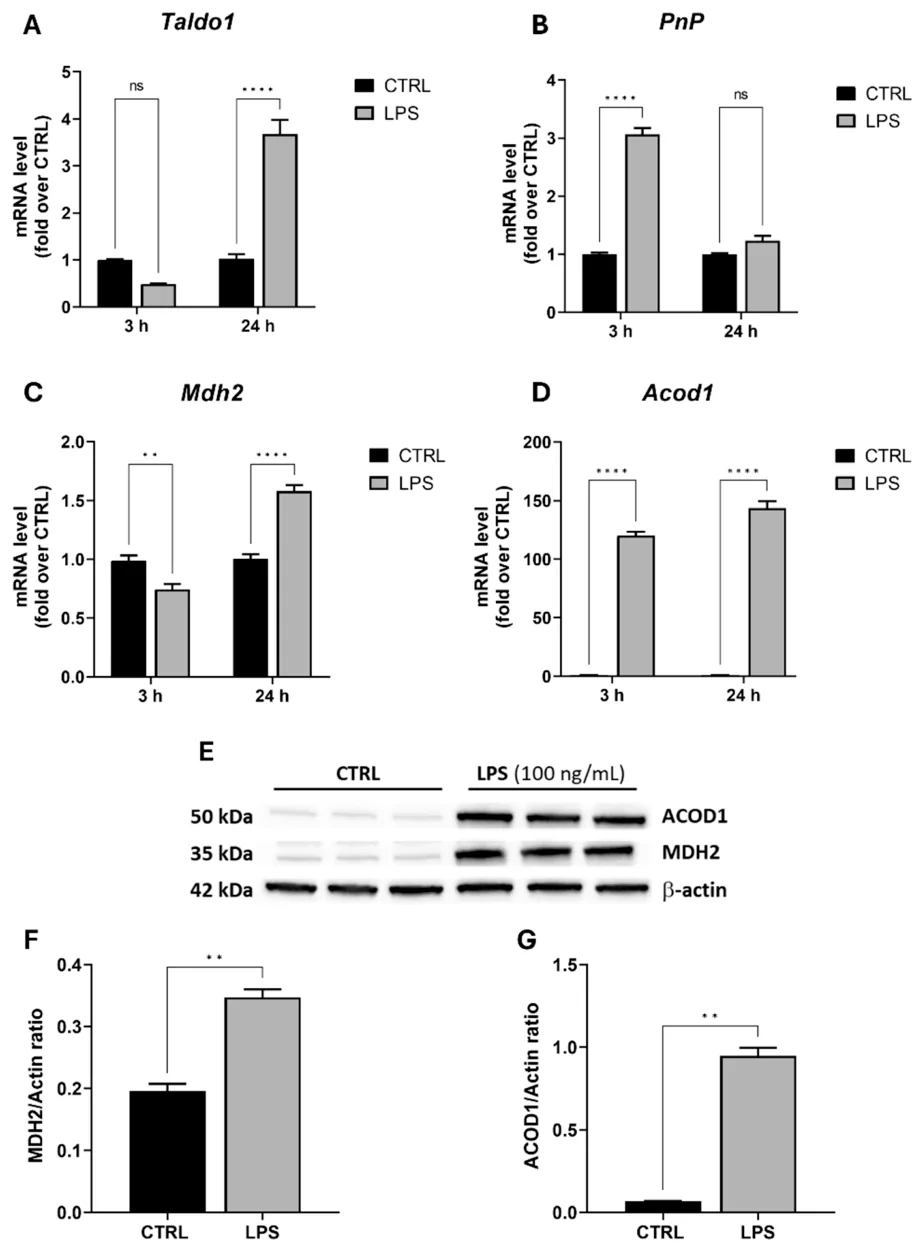
Expression of *Taldo1*, *Pnp*, *Mdh2*, and *Acod1* and protein abundance of MDH2 and ACOD1. BV-2 cells were treated for 3 h (**A**–**D**) and/or 24 h (**A**–**E**) with 100 ng/mL LPS. mRNA and protein levels were measured by real-time PCR and immunoblotting, respectively. Data are presented as mean ± SEM of at least three independent biological experiments performed on separate days (Table S2). Statistical analysis was performed using a two-way ANOVA, followed by Sidak’s post hoc test (**A**–**D**), and an unpaired *t*-test with Welch’s correction (**F**,**G**). ** *p* < 0.01; **** *p* < 0.0001. ns not significant.

Several metabolic enzymes were differentially modulated in the proteomic dataset. Proteomic analysis showed that Transaldolase 1 (TALDO1), an enzyme participating in the non-oxidative phase of the pentose phosphate pathway, was up-regulated after 24 h of LPS exposure. Consistent with these findings, *Taldo1* mRNA levels were significantly increased after 24 h, but were unchanged at 3 h (Figure 6A).

Moreover, the proteomic dataset showed increased purine nucleoside phosphorylase (PNP) abundance following 24 h LPS. Interestingly, our targeted validation analysis revealed higher *Pnp* mRNA levels at 3 h compared with control cells, but no significant differences were observed at 24 h. The difference between transcript and protein timing may reflect a transient transcriptional response, but an independent protein-level validation study is required (Figure 6B).

The proteomic analysis also identified upregulation of malate dehydrogenase 2 (MDH2), an enzyme in the tricarboxylic acid cycle (TCA). Immunoblotting confirmed increased MDH2 protein abundance after 24 h of LPS exposure (Figure 6E,F). On the other hand, *Mdh2* mRNA was reduced at 3 h and increased at 24 h, suggesting a differential effect of LPS on this enzyme at different time points of microglial activation (Figure 6C).

Aconitate decarboxylase 1 (ACOD1, also known as IRG1) was among the most differentially expressed proteins identified in the proteomic dataset.

*Acod1* mRNA levels were markedly and significantly increased at both (3 h and 24 h). Immunoblotting confirmed higher ACOD1 protein abundance (Figure 6D,E,G).

Since proteomic profiling was confined to 100 ng/mL LPS for 24 h, we next performed a separate targeted concentration-response analysis to examine whether increasing LPS exposure was associated with changes in cellular MTT-reducing capacity, oxidative status, antioxidant markers, and inflammatory mediators.

### 2.3. Effect of Increasing LPS Concentrations on Cellular Metabolic Activity

**Figure 7:**
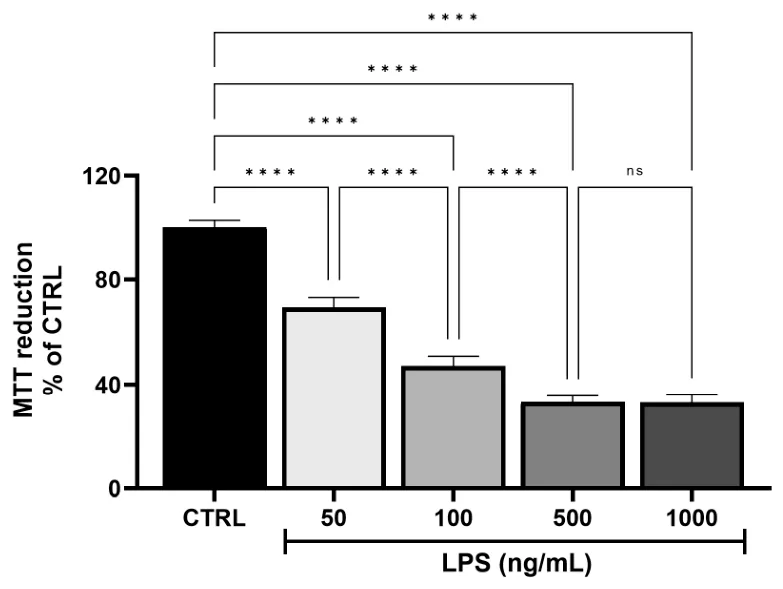
Effect of LPS on MTT-reducing capacity. BV-2 cells were treated with LPS at increasing concentrations (50, 100, 500, 1000 ng/mL) for 24 h, and MTT reduction was assessed as described in Materials and Methods. Data are expressed as % of control cells (CTRL) and represent mean ± SEM from three independent biological experiments performed on separate days (*n* = 3). Statistical analysis was performed using one-way ANOVA followed by Tukey’s test. **** *p* < 0.0001; ns not significant.

BV-2 cells were exposed to increasing concentrations of LPS (50, 100, 500, and 1000 ng/mL) for 24 h, and cellular MTT-reducing capacity was evaluated. LPS significantly decreased MTT reduction compared with untreated control cells (Figure 7). This reduction followed a concentration-dependent trend, with a progressive decrease from 50 to 500 ng/mL and a plateau between 500 and 1000 ng/mL.

### 2.4. Effect of Different LPS Concentrations on ROS Production

**Figure 8:**
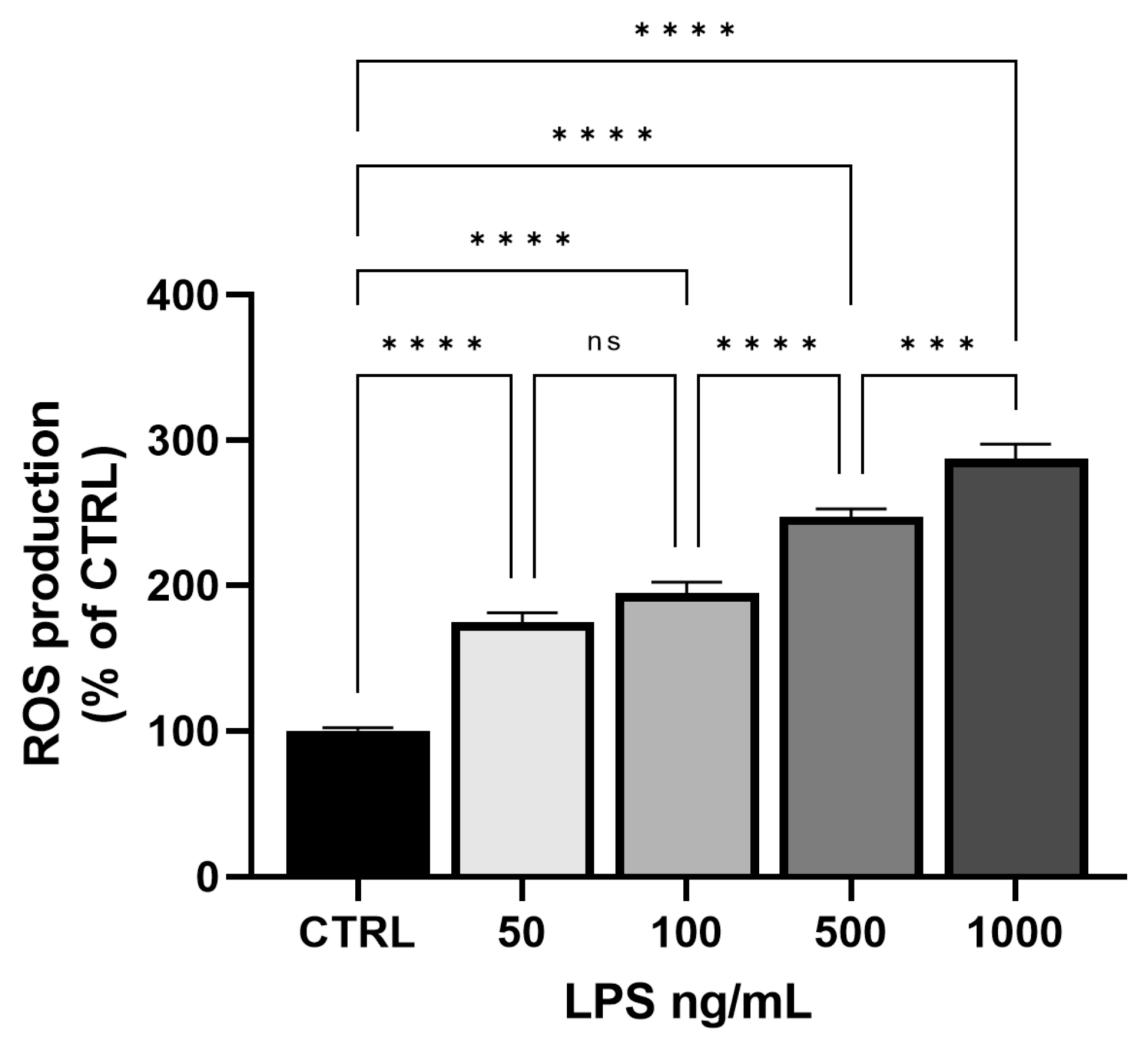
Effect of different LPS concentrations on ROS production. BV-2 cells were treated with increasing concentrations of LPS (50, 100, 500, 1000 ng/mL) for 24 h, and intracellular ROS levels were measured using the fluorescent probe DCFH-DA as described in Materials and Methods. Data are expressed as % of control cells (CTRL) and presented as mean ± SEM of three independent biological experiments performed on separate days (*n* = 3). Statistical analysis was performed using one-way ANOVA followed by Tukey’s test. *** *p* < 0.001; **** *p* < 0.0001; ns not significant.

To verify whether increasing LPS concentrations influenced intracellular oxidative status, intracellular ROS levels were measured using the DCFH-DA assay. As expected, the pro-inflammatory stimulus increased intracellular ROS levels at all concentrations tested compared with untreated cells, confirming the induction of oxidative stress in response to LPS (Figure 8). ROS production progressively increased from 50 to 1000 ng/mL LPS, despite no significant difference observed between 50 and 100 ng/mL. Although DCFH-DA does not discriminate among specific ROS, the results reflect the overall intracellular oxidative burden rather than individual ROS.

### 2.5. Effect of Different LPS Concentrations on Antioxidant Response-Related Gene and Protein Expression

**Figure 9:**
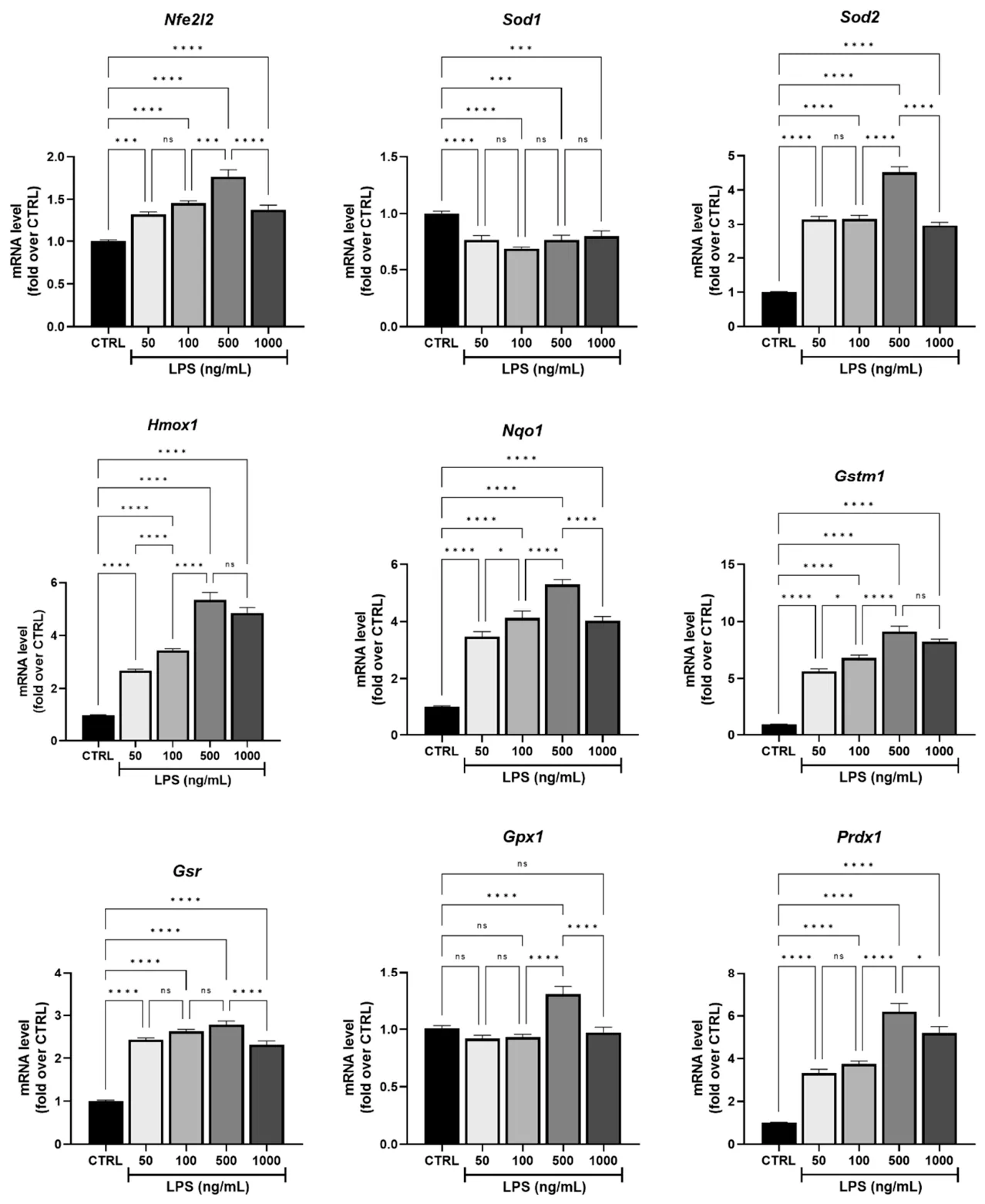
Gene expression of *Nfe2l2* transcription factor and selected antioxidant enzymes in BV-2 cells after exposure to different LPS concentrations. BV-2 cells were treated for 24 h with LPS at increasing concentrations (50, 100, 500, 1000 ng/mL), and gene expression of *Nfe2l2*, *Sod1*, *Sod2*, *Hmox1*, *Nqo1*, *Gstm1*, *Gsr*, *Gpx1*, and *Prdx1* was determined by real-time PCR, as reported in Materials and Methods. Data are expressed as fold change over CTRL. Bars represent mean ± SEM from three independent biological experiments performed on separate days (*n* = 3). Statistical analysis was performed using one-way ANOVA followed by Tukey’s test. * *p* < 0.05; *** *p* < 0.001; **** *p* < 0.0001; ns not significant.

To evaluate the activation of the cellular antioxidant machinery, the expression of *Nfe2l2* and its main antioxidant target enzymes was assessed by real-time PCR on BV-2 cells after 24 h exposure to increasing concentrations of LPS (Figure 9).

At concentrations from 50 to 500 ng/mL, LPS increased *Nfe2l2* and several antioxidant transcripts. Relative to 500 ng/mL, 1000 ng/mL significantly reduced *Nfe2l2*, Superoxide dismutase 2 (*Sod2*), *Nqo1*, Glutathione reductase (*Gsr*), and Glutathione peroxidase 1 (*Gpx1*) and Peroxiredoxin 1 (*Prdx1*) expression. The 500-versus-1000 ng/mL differences for heme oxygenase 1 (*Hmox1*) and Glutathione-S-transferase mu 1 (*Gstm1*) were not significant.

*Sod2* followed the non-linear pattern described above, whereas Superoxide dismutase 1 (*Sod1*) showed a modest reduction across LPS-treated groups and no significant differences among LPS concentrations.

**Figure 10:**
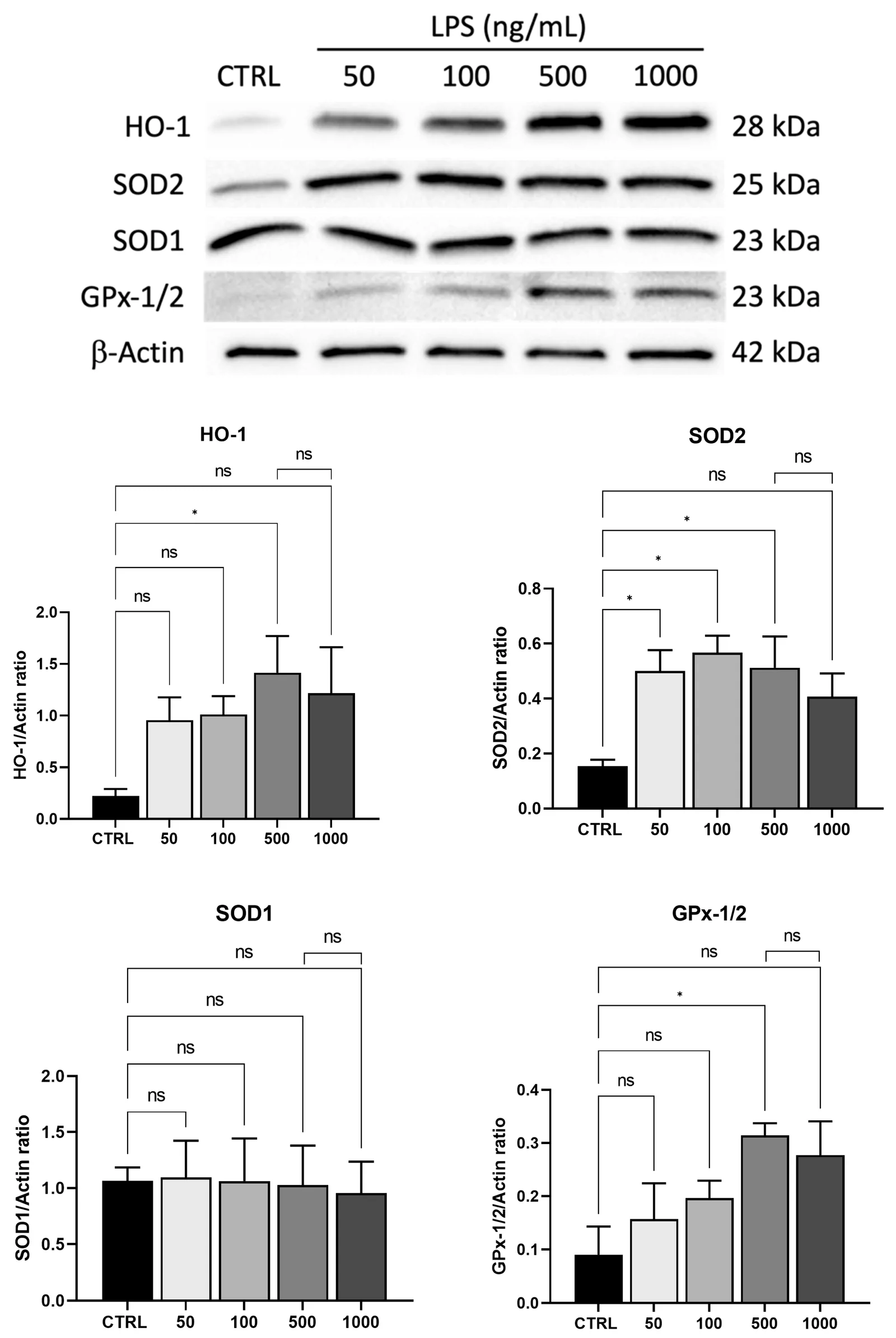
Protein levels of HO-1, SOD1, SOD2, and GPx-1/2 in BV-2 cells exposed to increasing concentrations of LPS. BV-2 cells were treated with LPS at increasing concentrations (50, 100, 500, 1000 ng/mL) for 24 h, and protein expression was assessed by Western blot using specific antibodies against HO-1, SOD1, SOD2, and GPx-1/2, as described in Materials and Methods. Data are presented as mean ± SEM of three independent biological experiments performed on separate days (*n* = 3). Statistical analysis was performed using one-way ANOVA followed by Dunnett’s test, and an unpaired *t*-test with Welch’s correction. * *p* < 0.05 vs. CTRL; ns not significant.

To further confirm these findings, protein levels of HO-1, SOD2, SOD1, and GPx-1/2 were analyzed by immunoblotting in cells exposed to increasing concentrations of LPS (Figure 10).

Overall, the protein expression profiles were consistent with the patterns observed at the transcriptional level, with HO-1, GPx-1/2, and SOD2 showing increased levels at intermediate LPS concentrations and reduced levels at the highest dose. However, the greater dispersion of the densitometric measurements prevented some differences from reaching statistical significance. In particular, at 1000 ng/mL the mRNA levels of *Sod2* and *Gpx1* were significantly lower than 500 ng/mL while this difference did not reach statistical significance at protein level. This difference between mRNA and protein results is not necessarily contradictory, because changes in transcript abundance do not invariably produce immediate or proportional changes in protein abundance. Protein levels are additionally influenced by translational regulation, protein turnover, and protein stability. Moreover, Western blot densitometry is subject to additional technical variability related to protein transfer, antibody detection, chemiluminescent development, and membrane-to-membrane differences, which may reduce the statistical power to detect relatively modest differences. In addition, *Gpx1* mRNA and the GPx-1/2 protein signal do not represent completely equivalent measurements, because the antibody detects both GPx isoforms. In contrast, SOD1 exhibited a slight, although not statistically significant, decrease across treatments, consistent with its mRNA expression pattern.

### 2.6. Effect of Different LPS Concentrations on Nitric Oxide (NO) Release, Inflammation-Related Gene, and Protein Expression

To compare the inflammatory response across LPS concentrations, the expression of inducible nitric oxide synthase (*Nos2*), NLR family pyrin domain containing 3 (*Nlrp3*), cyclooxygenase-2 (*Ptgs2*), *Il1b*, *Tnf*, interleukin-6 (*Il6*), and interleukin-4 (*Il4*) was assessed after 24 h of exposure to 50, 100, 500, or 1000 ng/mL LPS.

**Figure 11:**
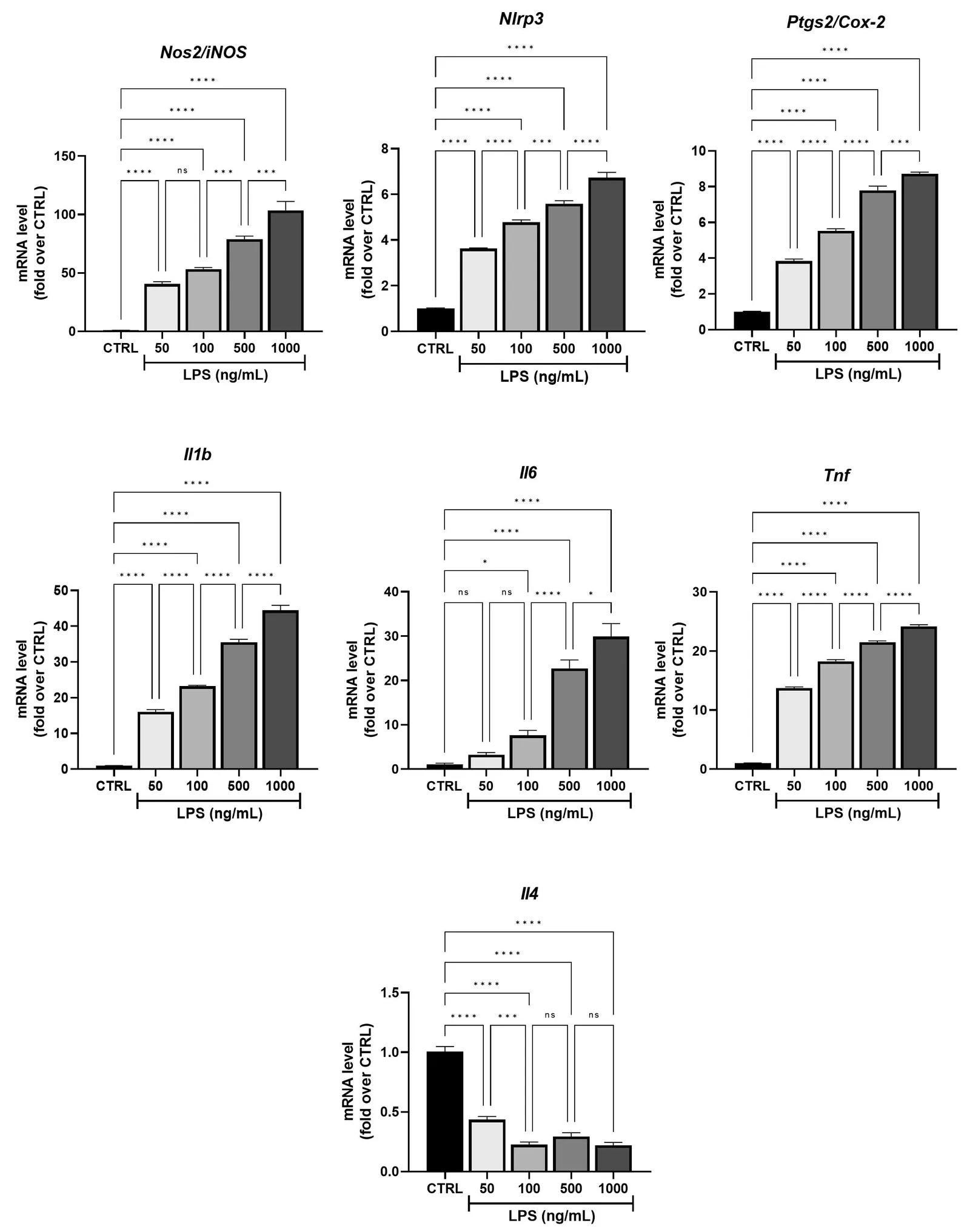
Expression of inflammation-related genes in BV-2 cells after exposure to different LPS concentrations. BV-2 cells were treated with LPS at increasing concentrations (50, 100, 500, 1000 ng/mL) for 24 h, and gene expression of *Nos2*, *Nlrp3*, *Ptgs2*, *Il1b*, *Tnf*, *Il6*, and *Il4* was determined by real-time PCR, as described in Materials and Methods. Data are expressed as fold change over CTRL. Bars represent mean ± SEM from three independent biological experiments performed on separate days (*n* = 3). Statistical analysis was performed using one-way ANOVA followed by Tukey’s test. * *p* < 0.05; *** *p* < 0.001; **** *p* < 0.0001; ns not significant.

**Figure 12:**
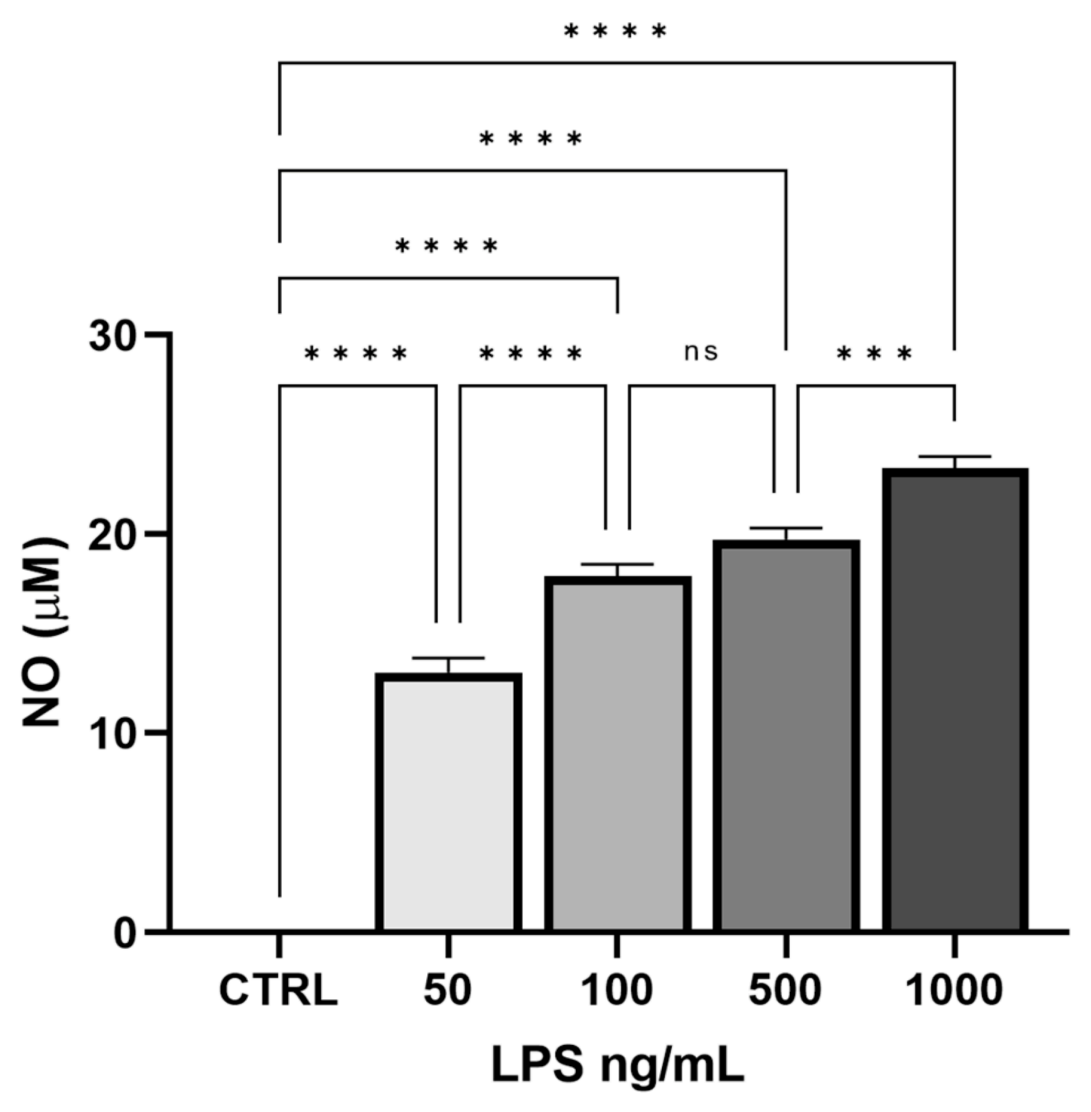
LPS-induced NO release by BV-2 cells. BV-2 cells were treated with LPS at increasing concentrations (50, 100, 500, 1000 ng/mL) for 24 h, and NO release was quantified by the Griess reagent in the culture medium, as reported in Materials and Methods. Data are represented as µM of NO. Bars represent mean ± SEM from three independent biological experiments performed on separate days (*n* = 3). Statistical analysis was performed using one-way ANOVA followed by Tukey’s test. *** *p* < 0.001; **** *p* < 0.0001; ns not significant.

Expression of pro-inflammatory genes increased across the LPS concentrations, including at 1000 ng/mL (Figure 11). NO release, assessed by the Griess assay, also increased with increasing LPS concentrations (Figure 12). Conversely, the expression of the anti-inflammatory cytokine *Il4* was reduced without a clear concentration-dependent pattern (Figure 11).

**Figure 13:**
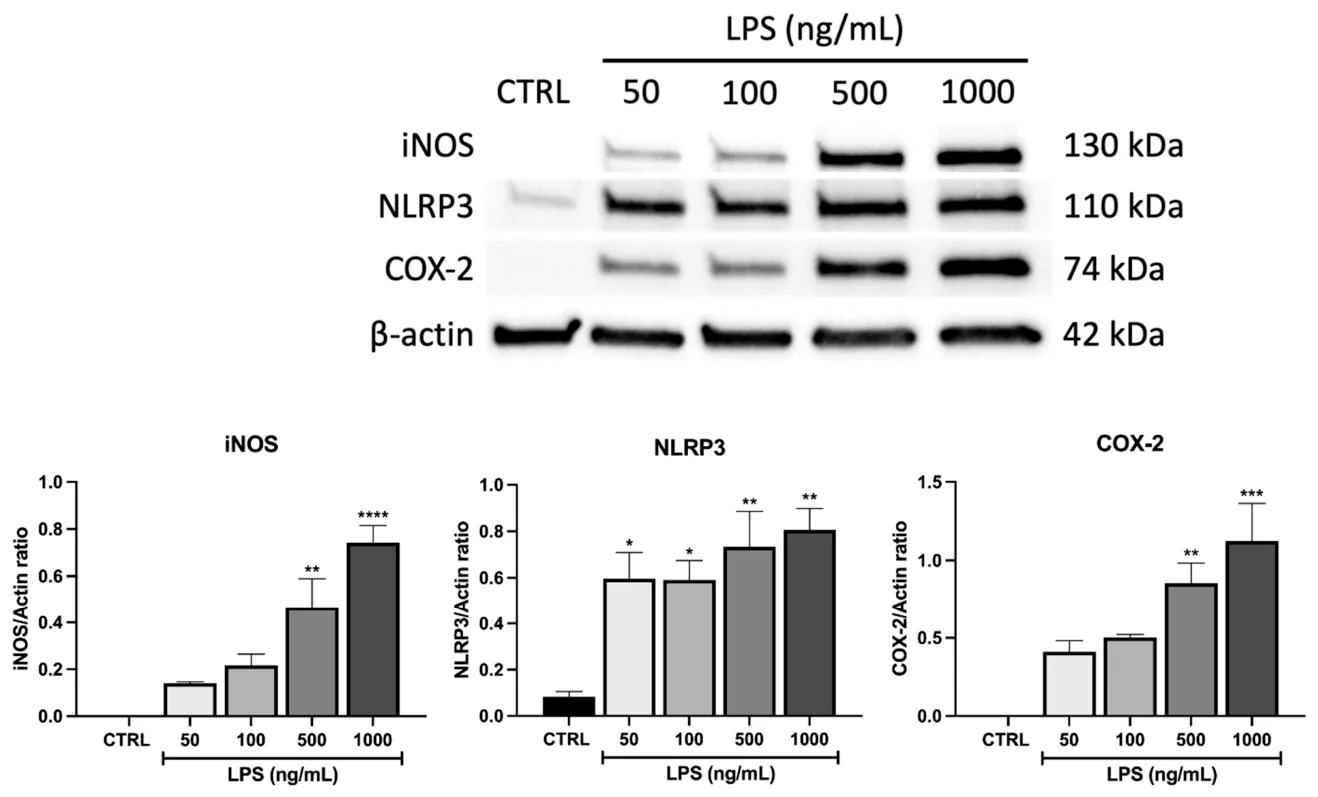
Protein levels of iNOS, NLRP3 and COX-2 in BV-2 cells exposed to increasing concentrations of LPS. BV-2 cells were treated for 24 h with LPS at different concentrations (50, 100, 500, 1000 ng/mL), and protein expression was assessed by Western blotting using specific antibodies against iNOS, NLRP3, and COX-2, as described in Materials and Methods. Data are presented as mean ± SEM of three independent biological experiments performed on separate days (*n* = 3). Statistical analysis was performed using one-way ANOVA followed by Dunnett’s test. * *p* < 0.05; ** *p* < 0.01; *** *p* < 0.001; **** *p* < 0.0001 vs. CTRL.

Finally, protein abundance of the inflammatory markers iNOS, NLRP3, and COX-2, was analyzed by immunoblot (Figure 13). A concentration-related increase in the protein abundance of all three inflammatory markers was observed in agreement with the expression data. iNOS and COX-2 levels were significantly increased only at the two highest LPS concentrations (500 and 1000 ng/mL), whereas NLRP3 abundance was significantly elevated at all concentrations tested. The increase in iNOS abundance was broadly consistent with the progressive rise in NO release shown in Figure 12. However, NO release was already significantly increased at 50 and 100 ng/mL, whereas the increase in iNOS protein reached statistical significance only at 500 and 1000 ng/mL, suggesting that the functional response measured in the culture medium may be detectable even when changes in protein abundance are relatively modest.

In conclusion, the targeted dose–response analyses showed different regulatory patterns for inflammatory and antioxidant markers. Pro-inflammatory mediators and oxidative markers remained elevated or increased as the LPS concentration rose, whereas the expression of several antioxidant genes increased up to 500 ng/mL and was significantly lower at 1000 ng/mL.

## 3. Discussion

The present study combines proteomic profiling of BV-2 microglial cells at a predefined reference condition (100 ng/mL LPS for 24 h) with targeted molecular and functional analyses. The proteomic and validation data characterize structural, trafficking, lysosomal, metabolic, and redox-related remodeling at this condition. The separate concentration-response experiments then show that selected inflammatory and antioxidant responses do not change in parallel as LPS exposure increases.

Proteomic analysis identified 89 differentially expressed proteins after LPS exposure, involving cytoskeletal organization, clathrin-mediated endocytosis, lysosomal activity, metabolic pathways, inflammatory signaling, and antioxidant responses. This broad pattern indicates that LPS-induced activation of BV-2 is accompanied by systems-level remodeling beyond the induction of classical inflammatory mediators.

Cytoskeletal remodeling was supported by reduced VIM and GSN abundance. VIM participates in intermediate-filament organization and has been linked to microglial activation and inflammasome regulation [27,28,29], whereas GSN regulates actin dynamics and has been reported to limit LPS-induced inflammatory signaling [30,31]. The reductions observed after 24 h may therefore reflect a later structural state of activated BV-2 cells rather than the initial triggering of inflammation. Together with the molecular validation, these findings define part of the cellular context in which sustained LPS signaling occurs, but their functional contribution to inflammatory and oxidative burden requires direct perturbation studies.

CME is one of the major routes for the internalization of various cargo molecules from the plasma membrane into the cytoplasm [32,33], including TLR4 and CD14 receptors [7,34]. DAB2, an endocytic adaptor protein involved in receptor selection for endocytosis [35,36], and CLTA, a component of the clathrin vesicles, have both been implicated in the TLR4 internalization and downstream signaling while clathrin-mediated endocytosis contributes to TLR4 internalization and downstream signaling [37,38]. The reduction of DAB2 and CLTA supports remodeling of the CME machinery. The present findings raise the hypothesis that altered trafficking may affect the duration or compartmentalization of LPS signaling. This mechanism was not tested directly, but it provides a potential link between structural remodeling and the inflammatory burden to which antioxidant pathways must adapt.

Another important aspect emerging from this study is the delayed activation of lysosomal pathways. While the expression of *Atp6v1e1*, *Ctsa*, and *Gusb* was unchanged or reduced during the early phase of LPS exposure, their levels increased after 24 h, in agreement with the proteomic data. In particular, ATP6V1E1 is a component of the V-ATPase complex, the proton pump responsible for lysosomal acidification [39], while CTSA and GUSB are lysosomal hydrolytic enzymes involved in the degradation of glycoproteins and glycosaminoglycans, respectively [40,41]. They all contribute to intracellular degradative processes. Interestingly, lysosomes are central not only to degradative processes but also to immune signaling, autophagy, metabolic adaptation, and redox regulation [39,42,43,44]. Therefore, the late induction of lysosomal-associated proteins may reflect increased turnover of damaged components, enhanced degradative demand, and the need to maintain cellular homeostasis under prolonged inflammatory activation.

The proteomic and targeted analyses also identified changes in *Taldo1*, *Pnp*, *Mdh2* mRNA and MDH2 protein, and *Acod1* mRNA and ACOD1 protein, consistent with immunometabolic remodeling. ACOD1 catalyzes the production of itaconate from cis-aconitate, thereby connecting TCA cycle remodeling with immunoregulatory and antioxidant signaling [45,46]. Evidence from other microglial models suggests that ACOD1 can influence NRF2-related signaling [47,48]. The early and sustained upregulation of *Acod1* observed in our study suggests that the itaconate pathway is rapidly engaged after LPS stimulation and may participate in a negative feedback response aimed at limiting excessive inflammation and supporting antioxidant adaptation. In the present study, however, ACOD1 induction and the IPA prediction of NRF2 activation are associative findings. They identify candidate links between metabolic and redox adaptation but do not demonstrate that ACOD1 drives the antioxidant response. The decrease in MTT reduction should not be regarded as contradictory to the increased abundance of MDH2 and ACOD1. Protein abundance does not directly report enzymatic activity or TCA-cycle flux, and ACOD1-mediated conversion of cis-aconitate to itaconate is itself a feature of TCA-cycle remodeling. Moreover, MTT reduction reflects composite cellular NAD(P)H-dependent reductive activity and should not be interpreted as a specific measure of mitochondrial respiratory-chain function or as a direct measure of cell viability [49]. Thus, lower MTT reduction may coexist with compensatory accumulation of selected metabolic enzymes and may reflect altered redox metabolism, mitochondrial stress, or fewer and/or metabolically less active cells. Direct respirometry, mitochondrial membrane-potential measurements, cell-death assays, and isotope-tracing studies would be required to distinguish these possibilities.

A central finding of this study is that the antioxidant and inflammatory responses showed distinct concentration-dependent patterns. At low-to-intermediate LPS concentrations, BV-2 cells exhibited increased expression of *Nfe2l2* and several antioxidant genes and proteins. This pattern is consistent with the activation of an adaptive antioxidant program and with the IPA prediction of NRF2 activation. Intracellular ROS levels significantly and progressively increased following LPS exposure. The inflammatory response displayed a different pattern. The expression of pro-inflammatory mediators, including *Nos2*, *Nlrp3*, *Ptgs2*, *Il1b*, *Tnf*, and *Il6*, remained upregulated across the concentration range, with NO production increasing progressively, as assessed by the Griess assay. By contrast, several antioxidant transcripts increased up to 500 ng/mL LPS and were significantly lower at 1000 ng/mL than at 500 ng/mL. This difference was observed for *Nfe2l2*, *Sod2*, *Nqo1*, *Gsr*, *Gpx1*, and *Prdx1*, whereas *Hmox1* and *Gstm1* did not differ significantly between these two concentrations. Western blot analyses were performed to assess the effects of treatment on the protein abundance of HO-1, SOD1, SOD2, and GPX1/2. Although the observed trends were consistent with those detected at the transcript level, the differences did not reach statistical significance. This discrepancy may be attributable to post-transcriptional and post-translational regulatory mechanisms, as well as to the inherent technical variability of Western blot analysis that could have reduced the statistical power to detect relatively modest differences in protein abundance.

The results, therefore, support an attenuation of selected antioxidant responses at the highest LPS concentration rather than a generalized failure of antioxidant defenses. This divergent behavior indicates that inflammatory activation and antioxidant adaptation follow different dose–response dynamics. This observation is important for the interpretation of the BV-2/LPS model itself, since different LPS concentrations may represent biologically distinct experimental states. At moderate LPS concentrations, inflammatory activation was is accompanied by increased expression of several NRF2-related antioxidant markers. In contrast, at 1000 ng/mL, persistent inflammatory activation was associated with lower expression of selected antioxidant transcripts compared to 500 ng/mL. These associations do not, however, establish a concentration threshold for failure of cytoprotective mechanisms or demonstrate a causal role for NRF2. The differential modulation of SOD1 and SOD2 may also be compatible with distinct regulation of cytosolic and mitochondrial antioxidant defenses. SOD2, the mitochondrial isoform, is typically upregulated in response to increased mitochondrial ROS production by pathways such as NF-κB and NRF2 [50,51], whereas SOD1 has a more constitutive role in cytosolic redox homeostasis [50,52].

Although mitochondrial ROS production and mitochondrial dysfunction have been reported in activated microglia [53,54], the present findings do not identify the subcellular source of ROS or directly demonstrate mitochondrial dysfunction. In particular, the observed reduction in MTT conversion reflects altered cellular reductive activity and cannot independently establish loss of viability or impaired mitochondrial function. Targeted mitochondrial and cell-viability assays would therefore be required to clarify these aspects.

Overall, the magnitude of inflammatory stimulation should be carefully considered when using BV-2 cells to model neuroinflammatory responses or to test antioxidant and anti-inflammatory interventions.

The present findings should be interpreted in light of some limitations. First, although BV-2 cells offer clear advantages in terms of reproducibility, scalability, and experimental control, they remain an immortalized murine microglial cell line and only partially reproduce the molecular and functional phenotype of primary microglia [15,16]. Validation in primary microglia, human induced pluripotent stem cell-derived microglia, or in vivo models is therefore required.

Second, the proteomic profiling was performed only at 100 ng/mL LPS for 24 h; the data therefore do not define dose- or time-dependent proteome remodeling or permit direct correlation of proteomic changes across LPS concentrations. Third, not every proteomic target was independently validated at the protein level. Future studies are therefore required to define the exact relationship among inflammatory burden, metabolism, and antioxidant adaptation.

## 4. Materials and Methods

### 4.1. Chemicals

Dulbecco’s modified Eagle’s medium (DMEM), penicillin/streptomycin, L-glutamine solution, phosphate-buffered saline (PBS), 3-(4,5-dimethylthiazol-2-yl)-2,5-diphenyltetrazolium bromide (MTT), 2′,7′-dichlorodihydrofluorescein diacetate (DCFH-DA), LPS from Escherichia coli serotype O127:B8, primers for real-time polymerase chain reaction, RIPA buffer, sodium pyro-phosphate, phenylmethylsulfonyl fluoride, dimethyl sulfoxide (DMSO), 2X Laemmli Buffer and all other chemicals of the highest analytical grade were purchased from Merck Italia (Milan, Italy). *DC* Protein Assay Kit, 10% Tween 20 solution, 10x Tris/Glycine buffer, 10× Tris/Glycine/SDS buffer, and 10× TBS were purchased from Bio-Rad (Hercules, CA, USA). Low-endotoxin fetal bovine serum (low-endotoxin FBS) was purchased from Euroclone (Milan, Italy). RNeasy Mini Kit was from Qiagen (Hilden, Germany). iScript™ cDNA Synthesis Kit was purchased from Bio-Rad (Hercules, CA, USA). Excel-Taq FAST qPCR SybrGreen (no ROX) was purchased from SMOBIO Technology, Inc. (Hsinchu City, Taiwan).

### 4.2. Cell Culture and Treatments

BV-2 murine microglial cells were kindly provided by Prof. Elisabetta Blasi (University of Modena and Reggio Emilia, Italy). BV-2 cells were cultured in DMEM supplemented with 10% (*v*/*v*) low-endotoxin FBS, 2 mM L-glutamine, 50 U/mL penicillin, and 50 μg/mL streptomycin, and maintained in a humidified incubator with 5% CO_2_ at 37 °C as reported in [21]. Cells were treated, in DMEM supplemented with 1% (*v*/*v*) low-endotoxin FBS, with LPS at different concentrations (50, 100, 500, 1000 ng/mL) for 24 h. In particular, proteomic and immunoblotting validations were performed with 100 ng/mL LPS for 24 h. For gene expression analysis in the context of proteomic validation, an additional 3 h time point was included to distinguish early from sustained transcriptional responses.

### 4.3. MTT Assay

Cellular MTT-reducing capacity was assessed as described previously [20]. BV-2 microglial cells were seeded in 96-well plates at a density of 1.0 × 10^4^ cells/well. To evaluate the effects of LPS on MTT-reducing activity, cells were treated as previously described. At the end of the treatments, cells were incubated with MTT solution (0.5 mg/mL) for 30 min at 37 °C. The solution was then replaced with 200 µL DMSO to solubilize the resulting formazan crystals. Absorbance was recorded at 570 nm using a multilabel plate reader (VICTOR3 V Multilabel Counter; Perkin-Elmer, Wellesley, MA, USA). MTT reduction was expressed as a percentage relative to untreated control cells.

### 4.4. DCFH-DA Assay

The intracellular production of ROS in BV-2 microglial cells was measured using the fluorescent probe DCFH-DA. Cells were seeded in 96-well plates at a density of 1.0 × 10^4^ cells per well and treated as previously described. They were then incubated with 10 μM DCFH-DA, prepared in phenol red- and FBS-free DMEM, for 30 min at 37 °C in the dark. After incubation, the probe was removed and replaced with PBS. Fluorescence intensity was measured using a VICTOR3 V Multilabel Counter (Perkin-Elmer, Wellesley, MA, USA), with excitation and emission wavelengths set at 485 nm and 535 nm, respectively. ROS levels were normalized on protein content. Briefly, PBS was replaced by RIPA buffer supplemented with a mammalian Protease Inhibitor Cocktail (1:100 dilution) and phenylmethylsulfonyl fluoride (10 mg/mL). Cell lysis was performed for 30 min on ice, followed by a freeze–thaw cycle. Total protein extract was collected and quantified using the Bio-Rad *DC* Protein Assay (BIO-RAD, Hercules, CA, USA).

Normalized ROS levels were expressed as a percentage relative to untreated control cells.

### 4.5. Griess Assay

NO release from cell culture was evaluated by measuring nitrite accumulation in the culture medium using the Griess reagent, as previously described [19]. BV-2 microglial cells were plated in 24-well plates at a density of 4.2 × 10^4^ cells per well and exposed to LPS as previously described.

At the end of the treatment, in order to remove debris and remaining cells, the culture media were centrifuged at 300× *g* for 10 min at 4 °C. Fifty microliters of each clarified supernatant were combined with an equal volume of Griess reagent in a 96-well microplate and incubated for 15 min. Absorbance was recorded at 540 nm using a multilabel microplate reader (TECAN SPARK; Tecan Group Ltd., Männedorf, Switzerland). Nitrite concentrations were calculated by comparison with a standard curve of NaNO_2_ (1, 2, 5, 10, 20, 40, 80, 120 μM).

### 4.6. Real-Time Polymerase Chain Reaction (Real-Time PCR)

For gene expression analysis, BV-2 cells were seeded into 6-well plates at a density of 2.0 × 10^5^ cells per well. After treatments, cells were rinsed with ice-cold PBS, and the total RNA was isolated using the RNeasy Mini Kit (Qiagen, GmbH, Hilden, Germany), according to the manufacturer’s protocol. RNA concentration and purity were determined using a NanoVue spectrophotometer (GE Healthcare, Milan, Italy). One microgram of RNA was then reverse-transcribed into cDNA using the iScript cDNA Synthesis Kit (BIO-RAD, Hercules, CA, USA). Real-Time PCR was performed in a 10 µL reaction volume, containing 5 µL of Excel-Taq FAST Q-PCR SybrGreen (SMOBIO Technology, Inc., Hsinchu City, Taiwan), 2.5 µL (12.5 ng) of cDNA, and 0.4 µL of each primer (400 nM final concentration), as detailed in (https://doi.org/10.3390/ijms242316601). Primer sequences are provided in Table 2. Amplification was carried out on the CFX Connect™ thermal cycler (BIO-RAD, Hercules, CA, USA), with an initial denaturation at 95 °C for 30 s, followed by 40 cycles of 5 s at 95 °C and 30 s at 60 °C. Melt curve analysis was used to confirm the specificity of amplification. Gene expression was normalized to the reference genes *Gapdh* and *Rpl38*, and analyzed using the 2^−ΔΔCT^ method, with untreated cells serving as the control.

### 4.7. Western Blotting

For protein expression analysis, BV-2 cells were seeded into 6-well plates at a density of 2.0 × 10^5^ cells per well. Cells were treated as previously described, then washed with ice-cold PBS and lysed using RIPA buffer supplemented with a mammalian Protease Inhibitor Cocktail (1:100 dilution) and phenylmethylsulfonyl fluoride (10 mg/mL) to preserve total protein content. The resulting lysates were quantified using the Bio-Rad *DC* Protein Assay (BIO-RAD, Hercules, CA, USA). Subsequently, samples were combined with an equal volume of 2X Laemmli Buffer (Sigma-Aldrich/Merck, Saint Louis, MO, USA), boiled for 5 min at 96 °C, then subjected to SDS-PAGE on 4–20% polyacrylamide gradient gels (BIO-RAD, Hercules, CA, USA). Proteins were subsequently transferred to nitrocellulose membranes (0.45 μm, BIO-RAD, Hercules, CA, USA) using Tris-glycine-methanol buffer at 110 V for 90 min.

Membranes were blocked with EveryBlot Blocking Buffer (BIO-RAD, Hercules, CA, USA) and incubated overnight at 4 °C on a 3D shaker with primary antibodies targeting iNOS (#13120), COX-2 (#12282), NLRP3 (#15101), HO-1 (#43966), ACOD1 (#19857), Gelsolin (#12953), Dab2 (#12906), MDH2 (#11908), and Vimentin (#5741) (Cell Signaling Technology, Beverly, MA, USA) at a 1:1000 dilution. Additional antibodies included GPx-1/2 (sc-30147), SOD-1 (sc-11407), and SOD-2 (sc-30080) (Santa Cruz Biotechnology, Dallas, TX, USA), all at 1:500, and anti-β-actin (A5441, Sigma-Aldrich–Merck, St. Louis, MO, USA) at a dilution of 1:5000, used as a loading control.

Following incubation with the appropriate secondary antibodies, signals were developed using Clarity™ Western ECL Substrate (BIO-RAD, Hercules, CA, USA). Band intensity and densitometric analysis were performed using ImageLab software version 5.2 (BIO-RAD, Hercules, CA, USA).

### 4.8. Proteomic Analysis

For proteomic analysis, BV-2 cells were seeded into 6-well plates at a density of 2.0 × 10^5^ cells per well. Cells were exposed to 100 ng/mL LPS for 24 h. At the end of the treatments, cells were collected and washed with PBS. After centrifugation (1000× *g* for 5 min), the resulting pellets were immediately frozen and stored at −80 °C until use. Two-dimensional electrophoresis (2DE) was performed as previously described [55]. Cellular protein extracts (200 µg) were loaded onto 18-cm IPG Blue Strips (Serva, Heidelberg, Germany) with a linear pH 3–10 gradient for first-dimension separation. The second dimension was carried out using 12% SDS-PAGE. Gels were stained with Ruthenium II tris (bathophenanthroline disulfonate) tetrasodium salt (RuBP; Cyanagen, Bologna, Italy), and images were acquired using the ImageQuant LAS4010 system (GE Healthcare, CA, USA). Image analysis was performed using SameSpot software (version 4.1, TotalLab, Newcastle Upon Tyne, UK). Spot volume ratios across conditions were calculated from the mean normalized spot volumes of three biological replicates. Statistical significance of differences in normalized spot volumes was determined using the ANOVA test implemented in the software. Protein spots displaying fold changes ≥ 1.5, *p* < 0.05, and *q* < 0.05 were selected for mass spectrometry identification.

### 4.9. Spot Digestion and Protein Identification

Selected protein spots were excised from the gels and subjected to in-gel trypsin digestion as previously described [55]. Tryptic peptides were analyzed by LC-MS/MS on an Ultimate 3000 RSLCnano system coupled to an Orbitrap Fusion Tribrid mass spectrometer (Thermo Fisher Scientific, Waltham, MA, USA), equipped with a nanoESI source (EASY-Spray NG) and operated in positive ion mode [56]. Raw files were processed in PEAKS Studio Xpro (v10.6 build 2020122; Bioinformatics Solutions Inc., Waterloo, ON, Canada) using the ‘correct precursor only’ setting. Spectra were searched against the UniProt/SwissProt database (supplemented with common contaminants), release 2023_04 (13 September 2023), restricted to Mus musculus (taxonomy ID: 10090) and comprising 17,178 reviewed protein sequences. and restricted to Mus musculus taxonomy (release [version/date], downloaded on [date]; [number] protein sequences). Peptide identifications were filtered using a peptide-level false discovery rate (FDR) of 0.1%. Protein identifications were accepted when the PEAKS protein confidence score was ≥20, expressed as −10log_10_(P). Identifications based on a single peptide were retained when they met these predefined criteria. Searches allowed non-specific cleavage at one peptide terminus, up to two missed cleavages, and a maximum of two variable PTMs per peptide. Mass tolerance limits were set to 10 ppm for precursor ions and 0.5 Da for fragment ions.

### 4.10. Bioinformatic Analysis

Volcano plot and statistical analysis of individual proteins were performed using GraphPad Prism 8 (GraphPad Software, Inc., La Jolla, CA, USA). Heatmap was generated using SRplot (https://www.bioinformatics.com.cn/SRplot, accessed on 16 March 2026) [57]. Proteins identified as differentially expressed were subjected to functional enrichment analysis using QIAGEN’s Ingenuity Pathway Analysis (IPA; QIAGEN, Redwood City, CA, USA; build version 321501M; content version 21249400). The analysis was used to characterize functional relationships among proteins and to identify enriched canonical pathways, upstream regulators, and molecular functions based on curated knowledge from the literature. Canonical pathways were evaluated according to their activation *z*-score, *p*-value, and ratio, with the latter representing the proportion of pathway-associated molecules found in the dataset. The activation *z*-score was used to predict the activation or inhibition of upstream regulators and molecular functions.

During data import, entries mapping to the same protein were resolved using the IPA duplicate-resolution function, which retained the entry with the highest fold-change value. Thus, one value was included for each unique protein in the Core Analysis. Because proteins identified in multiple spots may correspond to distinct proteoforms or post-translationally modified species, individual spot-level fold changes do not necessarily reflect changes in total protein abundance.

### 4.11. Statistical Analysis

Each experiment was performed at least three times, and all values are represented as mean ± SEM. Data from real-time PCR used for proteomic validation were analyzed using a two-way analysis of variance (ANOVA), followed by Sidak’s multiple-comparison test. Immunoblotting data were analyzed using an unpaired *t*-test with Welch’s correction (GraphPad Prism 9.4.1, San Diego, CA, USA). For all other data, one-way ANOVA was used to compare differences among groups, followed by Dunnett’s test or Tukey’s multiple comparisons test (GraphPad Prism 9.4.1, San Diego, CA, USA). Values of *p* < 0.05 were considered statistically significant.

## 5. Conclusions

In conclusion, proteomic profiling at 100 ng/mL LPS for 24 h, together with targeted molecular analyses, characterized remodeling of inflammatory, immunometabolic, lysosomal, endocytic, cytoskeletal, and redox-related pathways in BV-2 cells. In separate concentration-response experiments, increasing LPS exposure was associated with persistent inflammatory activation and with lower expression of several antioxidant enzymes at 1000 ng/mL than at 500 ng/mL LPS. These data support an association between high inflammatory burden and attenuation of selected antioxidant responses. These findings refine the interpretation of the BV-2/LPS model indicating that different LPS concentrations may represent biologically distinct inflammatory states rather than interchangeable experimental conditions. Although further validation in primary microglia, human-derived models, or in vivo systems will be required, and functional studies targeting NRF2 and ACOD1/IRG1 are needed to define causal mechanisms, this integrated framework provides a useful basis for future investigations into the immunometabolic and redox regulation of microglial activation.

## Figures and Tables

**Table 1:** Upstream Regulators with significant z-scores derived from IPA analysis of differentially expressed proteins obtained in the LPS vs. CTRL comparison.

Upstream Regulator	Molecule Type	Activation Z-Score	*p*-Value	Target Molecules
NRF2	transcription regulator	2.92	2.74 × 10^−^^17^	Aldoa, Cct3, Chordc1, Clpp, Cops5, Cs, Eif2s1, Esd, Fth1, Glud1, Gsr, Hsp90b1, Il1a, Pdia3, Pdia4, Pgd, Prdx1, Rplp0, Ruvbl1, Taldo1, Usp14, Vcp
KLF6	transcription regulator	2.76	6.05 × 10^−6^	Aldoa, Gapdh, Hspa5, Il1a, Lgals3, Pgk1, Psme2, Taldo1
SIX2	transcription regulator	2.24	2.90 × 10^−6^	Aldh2, Aldoa, Hspd1, Il1a, Pgk1
PKM	kinase	2.24	1.78 × 10^−5^	Aldoa, Gapdh, Gsr, Pck2, Taldo1
INSR	kinase	2.21	9.76 × 10^−4^	Aldh2, Atp5f1a, Atp5f1b, Cs, Gapdh, Hspd1, Mcm7, Mdh2
IL15	cytokine	2.02	1.46 × 10^−6^	Aldoa, Arhgdib, Gapdh, Hspa8, Il1a, Pdia3, Pdia4, Pgd, Pgk1, Pnp, Taldo1
CGA	other	−2.00	5.07 × 10^−4^	Hsp90b1, Hspa5, Pdia3, Pdia4
RGS2	enzyme	−2.00	4.20 × 10^−4^	Il1a, Pgk1, Prdx1, Vim
MMP9	peptidase	−2.00	2.97 × 10^−3^	Dab2, Ifit3, Lgals3, Psme2, Vim
MAPK1	kinase	−2.20	1.25 × 10^−2^	Eif5a, Hspa5, Il1a, Pdia3, Vim
ATG5	other	−2.24	2.68 × 10^−5^	Aldoa, Atp6v1e1, Eef2, Gapdh, Rplp0, Rpsa
FMR1	translation regulator	−2.24	1.78 × 10^−4^	Acod1, Gsr, Il1a, Pgk1, Vim
TGFBR2	kinase	−2.236	1.56 × 10^−2^	Aldh2, Aldoa, Cs, Glud1, Il1a, Mdh2, Pgk1
RORC	ligand-dep nuclear recep	−2.425	6.78 × 10^−6^	Atp6v1e1, Ctsa, Fth1, Gusb, Il1a, Lgals3, Prdx1
NPC1	transporter	−2.425	1.67 × 10^−6^	Il1a, Pgk1, Prdx1, Vim

**Table 2:** Primers used for Real-Time PCR.

Gene	5′-Forward-3′	5′-Reverse-3′	Ref. Seq
*Hmox1*	CATGAAGAACTTTCAGAAGGG	TAGATATGGTACAAGGAAGCC	NM_010442
*Nqo1*	CCTTTCCAGAATAAGAAGACC	AATGCTGTAAACCAGTTGAG	NM_008706
*Gsr*	GTTCACACAGGTTAAGGAAG	TATTCAGATTCAGGCCCTTAG	NM_010344
*Gstm1*	CTGACTTTGAGAAGCAGAAG	AAGCAAGGAAATCCACATAG	NM_010358
*Gpx1*	GGAGAATGGCAAGAATGAAG	TTCGCACTTCTCAAACAATG	NM_008160
*Prdx1*	ATTATACGACTAGTCCAGGC	GTACAAGAGTTTCTTCTGGC	NM_011034
*Nfe2l2*	CATTCCCGAATTACAGTGTC	GGAGATCGATGAGTAAAAATGG	NM_010902
*Sod1*	CACTCTAAGAAACATGGTGG	GATCACACGATCTTCAATGG	NM_011434
*Sod2*	CCATTTTCTGGACAAACCTG	GACCTTGCTCCTTATTGAAG	NM_013671
*Nos2*	CCTCCTCCACCCTACCAAGT	CACCCAAAGTGCTTCAGTCA	NM_010927
*Ptgs2*	TGGGGTGATGAGCAACTATT	AAGGAGCTCTGGGTCAAACT	NM_011198
*Nlrp3*	GATGCTGGAATTAGACAACTG	GTACATTTCACCCAACTGTAG	NM_145827
*Tnf*	CCCCAAAGGGATGAGAAGTTC	CCTCCACTTGGTGGTTTGCT	NM_013693
*Il1b*	GTTCCCATTAGACAACTGCACTACAG	GTCGTTGCTTGGTTCTCCTTGTA	NM_008361
*Il6*	GTCTATACCACTTCACAAGTC	TGCATCATCGTTGTTCATAC	NM_031168
*Il4*	CTGGATTCATCGATAAGCTG	TTTGCATGATGCTCTTTAGG	NM_021283
*Acod1*	GATGATCCTGGATTCTCTGG	AAAATCCATGGAGTGAACAG	NM_008392
*Pnp*	CTTTGACTACAATGAGATACCC	TGACAGTGAGTATCCTTCATAC	NM_013632
*Atp6v1e1*	AAGATTTCCCTTTGGTGAAG	AAACCTTTATCTTGCGATCC	NM_007510
*Clta*	AAGTATCCGTAAGTGGAGAG	CGTCAATGTCGTTTACAAAG	NM_001080384
*Mdh2*	AATCTAGTTTGCGTTGATGG	TAAAGAAAATCAGAGCCTGC	NM_008617
*Ctsa*	CTTCTATGACAACAAAGACCC	GTGTGTCCTCATATCTATGTC	NM_001038492
*Dab2*	AGAAAAAGGTTGAAGAAGCC	AGAGATCCAGAAAGTTCGAC	NM_001102400
*Gusb*	ATTCAGATATCCGAGGGAAAG	TCCTCTGAGTAGGGATAGTG	NM_010368
*Taldo1*	GTGTATCCACAGAAGTTGATG	CTTTGTAAAGCTCGATGAGG	NM_011528
*Gsn*	CTTTGTGATCGAAGAGGTTC	CTTTTCCAACCCAGACAAAG	NM_001206367
*Vim*	GAACCTGAGAGAAACTAACC	GATGCTGAGAAGTCTCATTG	NM_011701
*Gapdh*	ACCACAGTCCATGCCATCAC	TCCACCACCCTGTTGCTGTA	NM_001289726
*Rpl38*	GAAGGATGCCAAGTCTGTCAA	GAGGGCTGGTTCATTTCAGA	NM_001048057

## Data Availability

The original contributions presented in this study are included in the article and Supplementary Material. Further inquiries can be directed to the corresponding authors.

## Supplementary Materials

The following supporting information can be downloaded at https://www.mdpi.com/article/10.3390/ijms27208981/s1.
