# Supplementary material for: Inflammatory Burden Shapes Antioxidant Adaptation and Immunometabolic Remodeling in LPS-Activated BV-2 Microglial Cells
Source: Int J Mol Sci. 2026 Oct 10;27(20):8981. doi: 10.3390/ijms27208981 (PMC13704404; doi:10.3390/ijms27208981)
Supplement: Supplementary file 1 [file ijms-27-08981-s001.zip › Table S2.pdf]

Table S2. Number of biological replicates used for real-time PCR validation of proteomic results

Gene	CTRL 3 h	LPS 3 h	CTRL 24 h	LPS 24 h
Vim	3	3	3	4
Gsn	3	3	3	4
Dab2	3	3	3	3
Clta	3	3	3	4
Atp6v1e1	3	3	3	3
Ctsa	3	3	3	3
Gusb	3	3	3	4
Taldo1	3	3	3	3
PnP	3	3	3	4
Mdh2	3	3	3	4
Acod1	3	3	3	4
